# An Enhanced, Real-Time, Low-Cost GNSS/INS Integrated Navigation Algorithm and Its Platform Design

**DOI:** 10.3390/s25072119

**Published:** 2025-03-27

**Authors:** Pengcheng Wang, Yuting Gao, Qingzhi Zhao, Yalong Wang, Feng Zhou, Dengxiong Zhang

**Affiliations:** 1 College of Geomatics, Xi’an University of Science and Technology, Xi’an 710054, China; wpengcheng@stu.xust.edu.cn (P.W.); gaoyuting17@gmail.com (Y.G.); 23210226122@stu.xust.edu.cn (Y.W.); 19991868953@163.com (D.Z.); 2 College of Geodesy and Geomatics, Shandong University of Science and Technology, Qingdao 266590, China; zhouforme@163.com

**Keywords:** global navigation satellite system (GNSS), inertial navigation system (INS), unmanned aerial vehicles (UAVs), integrated navigation, real-time

## Abstract

The integration of the global navigation satellite system (GNSS) and the inertial navigation system (INS) is a well-established method for achieving accurate positioning, especially in applications involving unmanned aerial vehicles (UAVs). UAVs are increasingly used across various fields, yet they face challenges such as the need for real-time processing and the impact of low-quality measurements from cost-effective devices. To address these challenges, we propose a velocity-constrained, enhanced, real-time, low-cost, GNSS/INS integrated navigation algorithm and design an algorithmic platform based on the open-source software KF_GINS. The algorithm supports loosely coupled integration of GNSS position data and raw inertial measurement unit (IMU) data, utilizing a 4G data transmission unit (DTU) for real-time data transmission and performing loosely coupled computations on the received data. Subsequently, we successfully applied this algorithm to low-cost integrated navigation devices, such as UAVs. We tested the algorithm platform using one set of vehicle-mounted data and six UAV datasets. Experimental results indicate that the algorithm platform effectively performs computations under various conditions, improving single-point positioning (SPP) accuracy by up to 15.38% horizontally and 6.78% vertically. These findings demonstrate the algorithm platform’s capability to significantly enhance the accuracy and stability of integrated navigation positioning for UAVs.

## 1. Introduction

In recent years, the application of multi-source integrated navigation has expanded into various fields, including military and civilian sectors, making it a widely researched topic [1,2]. Among these, its application in the field of unmanned aerial vehicles (UAVs) has gained significant attention [3]. This is primarily due to the critical roles UAVs play in national defense, disaster response, logistics, and media entertainment [4], as well as their immense potential in improving efficiency, reducing costs, and enhancing safety. Therefore, leveraging the complementary advantages of multi-source sensors to improve the navigation accuracy and stability of UAVs has become an essential measure for ensuring their safe operation [5,6]. Among multi-source integrated navigation systems, the combination of the global navigation satellite system (GNSS) and the inertial navigation system (INS) is the most widely adopted [7]. As a representative navigation system, GNSS provides absolute, autonomous, and long-term positioning with high accuracy and wide coverage. However, it is susceptible to external interference [8]. In contrast, INS is immune to external interference and achieves positioning through its IMU, but it suffers from internal errors that lead to divergence over time [9]. The complementary nature of these systems [10] allows INS to maintain navigation output during short-term GNSS signal losses, while GNSS corrects INS errors during signal availability, ensuring system accuracy and stability [11]. GNSS/INS integration can be implemented in three architectures: loosely coupled, tightly coupled, and deeply coupled [12,13]. The loosely coupled approach is simple and easy to implement but offers lower accuracy. The tightly coupled approach provides higher accuracy and anti-interference capabilities but is more complex and less stable. The deeply coupled approach delivers the highest accuracy and robustness but is challenging to implement due to its complexity [14].

Research on GNSS/INS integrated navigation involves multiple aspects, with a common focus on improving positioning accuracy and robustness under GNSS signal blockage conditions [15]. Dai et al. [16] proposed a GNSS/INS integrated navigation method using a recurrent neural network (RNN) to address GNSS signal loss. This method leverages the memory function of RNNs and the computational principles of INS to estimate errors, thereby achieving high-precision positioning solutions. Shi et al. [17] introduced a tightly coupled GNSS/INS post-processing smoothing method, which reinitializes integer ambiguities and processes raw GNSS observations using the Rauch–Tung–Striebel (RTS) smoothing algorithm. Their results demonstrate that this approach significantly enhances the accuracy of positioning and attitude determination. With the advancement of microelectronics technology, devices have become increasingly miniaturized, leading to extensive research on low-cost integrated navigation systems [18]. Chen et al. [19] proposed a method that integrates time-differenced GNSS carrier phase with INS measurements, incorporating non-holonomic constraints and odometer data to suppress divergence. This approach improves the navigation performance of low-cost systems in land vehicles. Vanek et al. [20] utilized a tightly coupled method with low-cost inertial sensors to assist in GNSS cycle slip detection. Compared to traditional methods, their approach increased the integer ambiguity fix rate by 19%. In terms of open-source software platforms, Chen et al. [21] released a GNSS/INS integrated navigation software based on MATLAB (version 9.4, MathWorks, Inc., Natick, MA, USA), supporting loosely coupled and tightly coupled post-processing solutions. This software significantly improves computational accuracy compared to standalone GNSS solutions. Niu et al. open-sourced the KF_GINS software [22,23], which implements loosely coupled GNSS/INS integration, providing beginners with rich learning resources. In the field of UAV navigation, Gyagenda N. et al. [24] summarized the historical development and strategies of UAV navigation. Chi et al. [25] analyzed the advantages of real-time PPP/INS tightly coupled navigation systems for UAVs, simulating and evaluating the system’s accuracy during GNSS signal outages. Solomon P. D. et al. [26] developed a technique for real-time estimation of GNSS delays in low-cost integrated navigation systems, using an extended Kalman filter to fuse delay measurements in real time and compensate for GNSS delays. Wu et al. [27] conducted an in-depth analysis of key technologies for low-cost sensor-integrated navigation in multi-rotor UAVs, systematically reviewing the development status, principles, and algorithms of low-cost devices in UAV navigation.

The algorithm proposed in this study is developed based on the open-source software KF_GINS. Building upon the loosely coupled extended Kalman filter (EKF) with GNSS position constraints, we incorporated Doppler velocity constraints and integrated them into a real-time low-cost GNSS/INS integrated navigation experimental platform. This platform is equipped with low-cost integrated navigation sensors and supports real-time transmission and processing of raw data. Using the experimental platform, we collected raw GNSS and IMU observation data from ground vehicle tests and multiple sets of rotorcraft UAVs experiments to conduct loosely coupled integration tests. The focus was on evaluating the impact of velocity constraints on the positioning accuracy of low-cost devices. By statistically analyzing the experimental results, we compared the positioning performance with and without velocity constraints for both vehicles and rotorcraft UAVs. Special attention was given to the performance improvement of the loosely coupled GNSS/INS system on UAVs when velocity constraints were applied. This paper describes the construction of a real-time, low-cost GNSS/INS integrated navigation algorithm platform. 

## 2. Materials and Methods

The Kalman filter is a widely adopted optimal estimation method that iteratively updates predictions and measurements to derive optimal estimates. It is known for its high computational efficiency and accuracy. In this study, the optimal estimation for GNSS/INS integrated navigation is designed based on the discrete Kalman filter [28]. The Kalman filter’s information update process consists of two steps: prediction and measurement updates. During the prediction step, the state and its covariance are calculated as follows:

(1) $${\hat{x}}_{k/k-1}=\Phi_{k/k-1}{\hat{x}}_{k-1}$$

(2) $$P_{k/k-1}=\Phi_{k/k-1}P_{k-1}\Phi_{k/k-1}^{T}+W_{k-1}Q_{k-1}W_{k-1}^{T}$$

where ${\hat{x}}_{k/k-1}$ represents the predicted state at time k, $\Phi_{k/k-1}$ is the state transition matrix from time k − 1 to k, ${\hat{x}}_{k-1}$ denotes the optimal state estimate at time k − 1, $P_{k/k-1}$ is the predicted covariance matrix at time k, $P_{k-1}$ is the optimal covariance matrix estimate at time k − 1, $W_{k-1}$ is process noise drive matrix, and $Q_{k-1}$ is the system noise covariance matrix.

The update step of the Kalman filter primarily relies on the Kalman gain matrix. The state vector and covariance matrix are updated using the following equations:

(3) $$K_{k}=P_{k/k-1}H_{k}^{T}/(H_{k}P_{k/k-1}H_{k}^{T}+R_{k})$$

(4) $${\hat{x}}_{k}={\hat{x}}_{k/k-1}+K_{k}(z_{k}-H_{k}{\hat{x}}_{k/k-1})$$

(5) $$P_{k}=(I-K_{k}H_{k})P_{k/k-1}{(I-K_{k}H_{k})}^{T}+K_{k}R_{k}K_{k}^{T}$$

In the equations, $K_{k}$ is the Kalman gain at time *k*, $H_{k}$ is the observation matrix at time *k*, $R_{k}$ is the observation noise covariance matrix, and $z_{k}$ is the measurement at time *k*.

Typically, the error-state Kalman filter is employed for loosely coupled integration, where the error state vector is defined as follows:

(6) $$\Delta x(t)=\left[\begin{array}{ccccccc} \Delta p & \Delta v & \phi & b_{g} & b_{a} & s_{g} & s_{a} \end{array}\right]$$

In the equation, Δp represents the position error vector, Δv is the velocity error vector, ϕ denotes the attitude error vector, $b_{g}$ and $b_{a}$ are the gyroscope bias error vector and accelerometer bias error vector, respectively, and $s_{g}$ and $s_{a}$ represent the gyroscope scale factor error vector and accelerometer scale factor error vector, respectively.

The system state equation can be expressed as a differential equation with respect to time as follows:

(7) $$\Delta \dot{x}(t)=F(t)\Delta x(t)+G(t)\omega (t)$$

By taking the first-order time derivative of each component of the vector Δx(t), the differential equations for position, velocity, and attitude can be derived. The coefficient matrices of the derivatives are then combined, and the bias and scale factor errors are modeled using a first-order Gauss–Markov process to obtain the coefficient matrix F(t), Based on the system state equation, the coefficient matrix G(t) for the noise vector can be determined.

### 2.1. Observation Equation with Position Constraints

The measurement can be expressed in error form as follows:

(8) $$\Delta z_{p}=H_{p}\Delta x+\varepsilon_{p}$$

Here, the observation error term z and the observation matrix H are specifically expressed under the same filtering state as follows:

(9) $$H={\left[\begin{array}{ccccccc} I_{3} & 0_{3} & \left(C_{b}^{n}l^{b}\times \right) & 0_{3} & 0_{3} & 0_{3} & 0_{3} \end{array}\right]}_{3\times 21}$$

(10) $$z={\left[\Delta S\right]}_{3\times 1}$$

In the above equation, $C_{b}^{n}$ is assigned using the latest inertial navigation system (INS) attitude, and $l^{b}$ is the lever arm vector.

The noise matrix for the observed values is expressed as follows:

(11) $$R={\left[R_{S}\right]}_{3\times 3}$$

Here, $R_{S}$ are the noise matrices for the observed position and are assigned using the variances of the current position.

### 2.2. Observation Equation with Position and Velocity Constraints

The measurement can be expressed in error form as follows:

(12) $$\left\{\begin{array}{l} \Delta z_{p}=H_{p}\Delta x+\varepsilon_{p}\\ \Delta z_{v}=H_{v}\Delta x+\varepsilon_{v} \end{array}\right.$$

Here, the observation error term z and the observation matrix H are specifically expressed under the same filtering state as follows:

(13) $$H={\left[\begin{array}{ccccccc} I_{3} & 0_{3} & \left(C_{b}^{n}l^{b}\times \right) & 0_{3} & 0_{3} & 0_{3} & 0_{3}\\ 0_{3} & I_{3} & H_{v3} & -(C_{b}^{n}l^{b}\times ) & 0_{3} & H_{v6} & 0_{3} \end{array}\right]}_{6\times 21}$$

(14) $$z={\left[\begin{array}{l} \Delta S\\ \Delta V \end{array}\right]}_{6\times 1}$$

In the above equation, $C_{b}^{n}$ is assigned using the latest inertial navigation system (INS) attitude, and $l^{b}$ is the lever arm vector.

The noise matrix for the observed values is expressed as follows:

(15) $$R={\left[\begin{array}{cc} R_{S} & \\ & R_{v} \end{array}\right]}_{6\times 6}$$

Here, $R_{S}$ and $R_{v}$ are the noise matrices for the observed position and velocity, respectively, and are assigned using the variances of the current position and velocity.

## 3. Experimental Setting and Data Collection

To validate the performance of the low-cost integrated navigation experimental platform proposed in this study and evaluate the accuracy of the Kalman filter with velocity constraints, data collection and solution analysis were conducted using this platform. Multiple sets of raw GNSS and IMU observation data were collected from different platforms, including one set of vehicle-mounted data and six sets of UAV-mounted data. The UAV used in the experiments was a DJI M350 RTK (Shenzhen Dajiang Innovation Technology Co., Ltd., Shenzen, China), while the vehicle was a small car. The data collection was conducted in May and September 2024. The vehicle-mounted data were collected from 11:12 to 12:13 on May 30, with a total duration of one hour. The UAV data collection took place from 09:44 to 13:05 on 26 September, comprising six sets of data, each with a duration of approximately 30 min. All data were collected in Lantian County, Xi’an, China, in a relatively open environment. 

**Figure 1:**
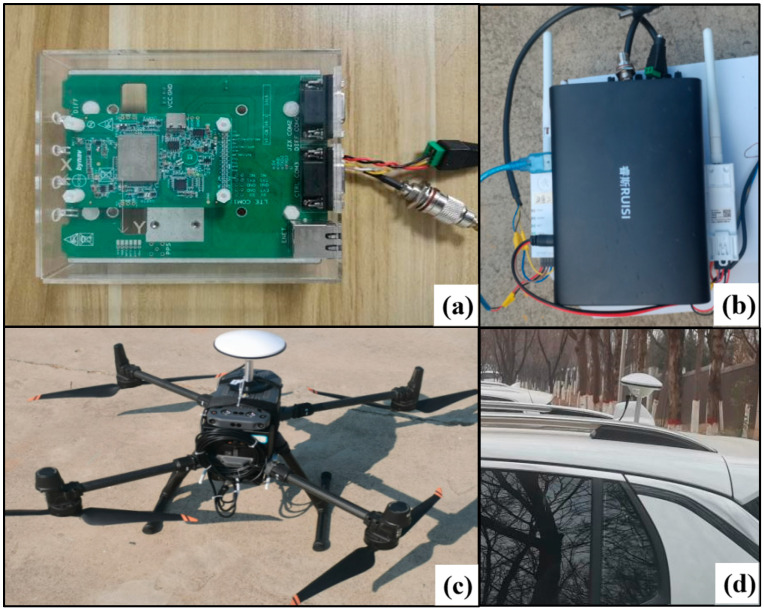
Diagram of experimental equipment and carriers (In the figure, (**a**–**d**) represent the integrated navigation device, power and data transmission equipment, the unmanned aerial vehicle (UAV), and the mobile vehicle, respectively).

The integrated navigation device used was the Beiyun M22 navigation board (Hunan Bynav Technology Co.,Ltd, Hunan, China), which incorporates a GNSS receiver chip and a MEMS IMU sensor. The detailed specifications are listed in Table 1, “Integrated Navigation Device Parameters”. The base station was a self-established station equipped with a BDStar M66 receiver (Beijing BDStar Navigation Co., Ltd., Beijing, China) and an HX-CSX627A antenna (Shenzhen Huaxin Antenna Technology Co., Ltd., Shenzhen, China). Vehicle-mounted data were received via a serial port, while GNSS and IMU raw observation data from the UAV were transmitted wirelessly using a DTU module. The GNSS raw observation data were sampled at 1 Hz, and the IMU output was sampled at 100 Hz. The platforms and equipment used in the experiments are shown in Figure 1, “Experimental Equipment and Platforms”. Figure 1a,b depict the integrated navigation device and data transmission module, respectively, while Figure 1c,d show the UAV platform and the vehicle platform, respectively.

**Figure 2:**
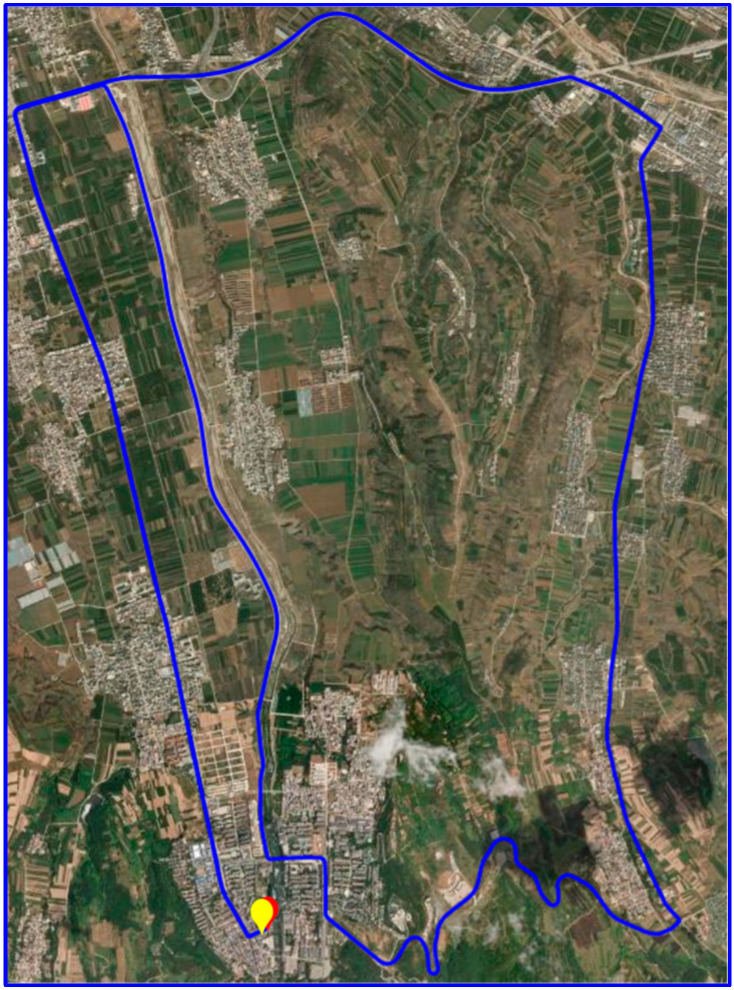
Trajectory diagram of the vehicle-mounted experiment (In the figure, the starting point is marked in yellow, while the endpoint is indicated in red).

**Figure 3:**
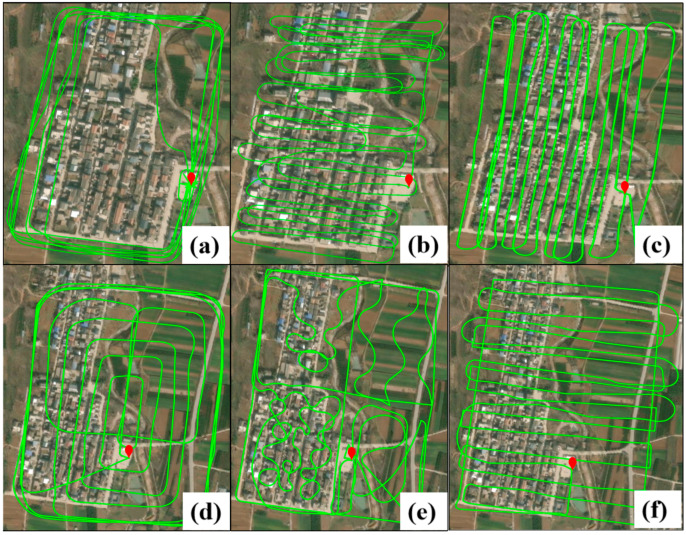
Six different trajectories diagrams of the airborne experiment. The red markers in the figure indicate the starting point and the ending point.

All data collection experiments were conducted in suburban areas with open environments, ensuring high-quality data acquisition. Figure 2 presents the trajectory of the vehicle-mounted dynamic experiment, while Figure 3 shows the trajectories of the UAV-mounted dynamic experiments. Figure 3a–f correspond to the six sets of UAV data collected at different times.

## 4. Results

In this section, we first analyze the IMU errors that require compensation, followed by an investigation of the changes in vehicular integrated navigation data before and after the incorporation of velocity constraints. Finally, a statistical analysis is conducted on six sets of UAV data to evaluate the impact of velocity constraints. The results demonstrate that the integration of velocity constraints leads to a noticeable improvement in the positioning accuracy of the integrated navigation system.

### 4.1. Error Compensation for Low-Cost IMUs

**Figure 4:**
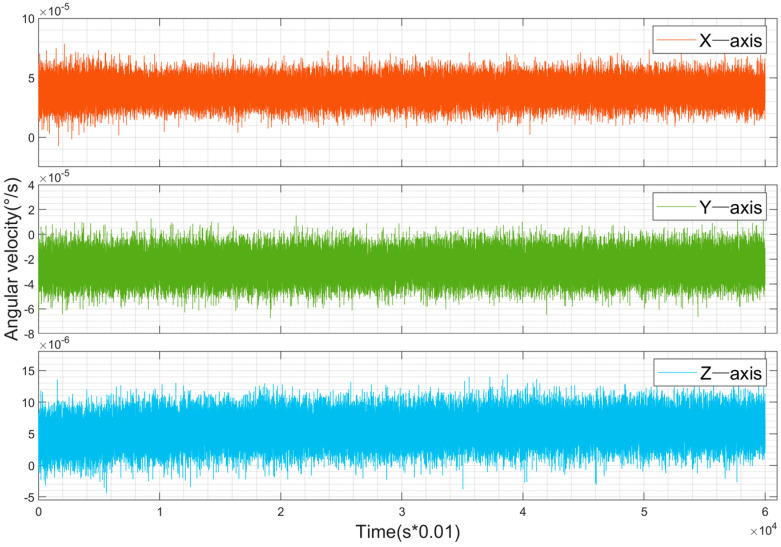
Raw gyroscope data from the static IMU.

**Figure 5:**
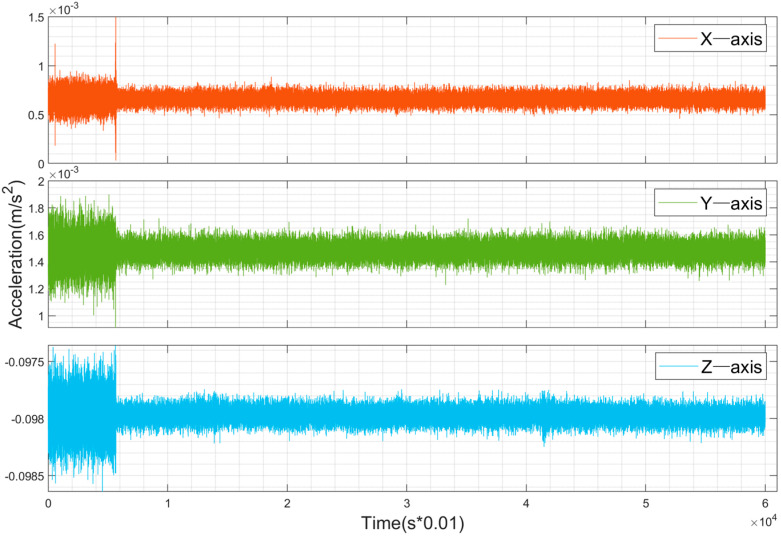
Raw accelerometer data from the static IMU.

The experimental platform in this study is equipped with a low-cost MEMS IMU for data acquisition. To evaluate the bias of the device in practical experiments, static data were collected by placing the device on a level surface. Figure 4 and Figure 5 show the raw observation data from the gyroscope and accelerometer, respectively, under static conditions. The data include the Earth’s rotation and were sampled at 100 Hz. From Figure 4, it can be observed that the outputs of the gyroscope in the static state are 14.10°/h, –9.23°/h, and 2.02°/h for the x, y, and z axes, respectively. This is due to the influence of the Earth’s rotation (15°/h) on the horizontal axes, while the true bias is close to that of the z-axis, approximately 2°/h. From Figure 5, it is evident that the accelerometer exhibits significant noise during the initial phase, which is caused by vibrations when the device is first placed at rest. In the static state, the outputs of the accelerometer are 0.067 m/s^2^, 0.148 m/s^2^, and -9.79 m/s^2^ for the x, y, and z axes, respectively. The z-axis output is approximately the negative value of the local gravitational acceleration, while the x and y axes represent the true bias. The measured biases of both the gyroscope and accelerometer require compensation during calculations.

### 4.2. Ground Vehicle Experiment Analysis

**Figure 6:**
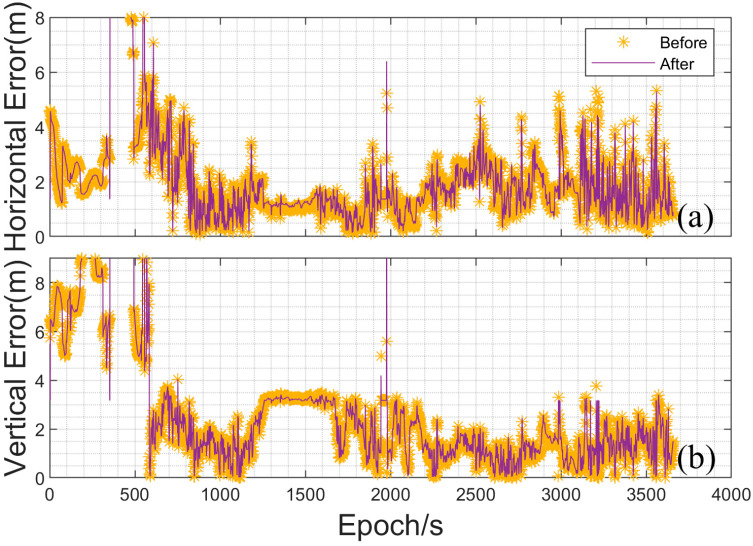
Horizontal and vertical direction errors of vehicle-mounted data (In the figure (**a**,**b**) are horizontal and vertical, respectively).

To evaluate the performance of the experimental platform in ground vehicle navigation, the vehicle-mounted data were processed and analyzed using both the original KF_GINS software and the proposed software platform. Given the overall duration of the data, the data processing strategy adopted was loosely coupled integration with single point positioning (SPP), using real-time kinematic (RTK) results as the reference truth for comparison. Figure 6 shows the deviation plots between the integrated navigation results before and after improvements and the RTK positioning results. Figure 6a depicts the horizontal deviation, where it can be observed that the overall trends before and after improvement show no significant difference, with the horizontal deviation of the SPP loosely coupled results mostly within 5 m. Further analysis reveals that at certain points with larger deviations, the improved experimental platform exhibits relatively smaller deviations. Figure 6b shows the vertical deviation, where, compared to the horizontal direction, the vertical error is larger during the first 10 min of data collection, although the deviation trends in both directions are consistent. Additionally, the mean of bias and root mean square error (RMSE) were calculated for both horizontal and vertical directions. In the horizontal direction, the bias improved from 2.11 m to 2.07 m, showing an overall improvement of 4 cm. The RMSE improved from 2.79 m to 2.73 m, with a 6 cm enhancement. In the vertical direction, the biases before and after improvement were 4.845 m and 4.85 m, respectively, while the RMSE remained at 4.95 m, indicating limited improvement in the vertical direction. Considering the inherent accuracy of SPP, the improved experimental platform achieves the expected performance in SPP loosely coupled integration and demonstrates enhanced accuracy compared to the original software.

### 4.3. UAV Onboard Experiment Analysis

**Figure 7:**
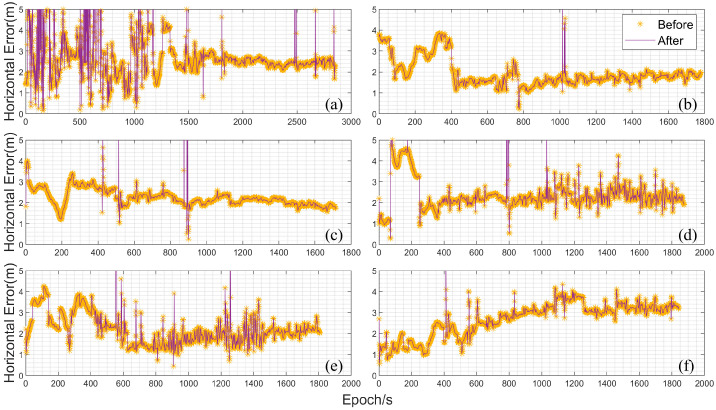
Horizontal direction errors of UAV-mounted data (In the figure (**a**–**f**) represent six sets of UAV data, respectively).

To further analyze and verify the computational accuracy and reliability of the improved experimental platform, multiple sets of UAV-mounted data were processed and analyzed before and after the improvements. The processing strategy adopted was again loosely coupled integration with SPP, using RTK results as the reference truth. Figure 7 shows the horizontal deviation plots for the UAV-mounted data, with Figure 7a–f representing the six sets of collected data. As shown in the figure, the duration of the data in Figure 7a is the longest, nearly 50 min, while the subsequent sets were collected for approximately 30 min each. Additionally, the initial phase of Figure 7a exhibits poor data quality with significant error fluctuations, which stabilize in the middle phase. The other datasets remain relatively stable during the flight phase, but all show a relatively large fluctuation between 200 s and 400 s, caused by the UAV’s takeoff. Figure 7b shows the smallest error after takeoff, with horizontal errors remaining within 2 m. Figure 7c–e maintain an accuracy of around 3 m, while Figure 7f starts with smaller errors but shows a significant increase over time. Overall, the differences before and after the improvements are not highly pronounced, but noticeable improvements can be observed at certain points. Both the original and improved loosely coupled results remain within 5 m, indicating that the improved experimental software platform can perform loosely coupled computations while maintaining accuracy.

**Figure 8:**
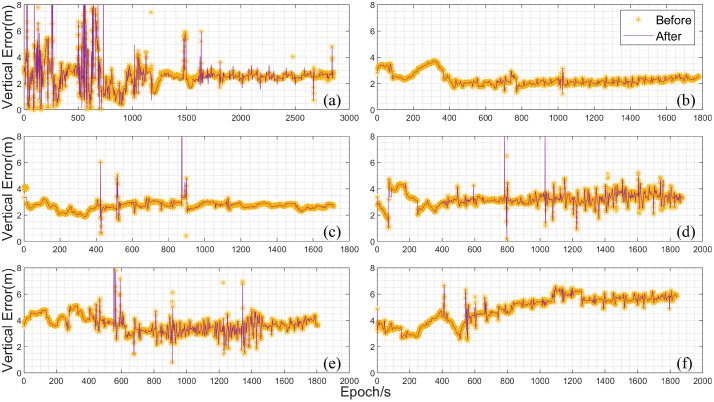
Vertical direction errors of UAV-mounted data (In the figure (**a**–**f**) represent six sets of UAV data, respectively).

In addition, the vertical errors of the UAV-mounted data were also analyzed through graphical plots. Figure 8 shows the vertical error plots for the UAV-mounted data, with the figure sequence corresponding to Figure 7. As can be seen from the figure, the vertical errors are generally larger compared to the horizontal errors. In Figure 8a, except for significant fluctuations in the first 1000 s, the errors stabilize around 3 m afterward. Figure 8b exhibits the best stability, with errors stabilizing around 2 mafter the UAV’s takeoff. Figure 8c,d show errors fluctuating around 3 m, while Figure 8f demonstrates the largest error variation, reaching approximately 6 m by the end of the data collection. It can be observed that the experimental platform used in this study performs loosely coupled computations relatively stably. However, the specific numerical changes before and after the improvements are not clearly visible in the error plots. Therefore, to specifically analyze the changes in bias before and after the improvements, the bias data from Figure 7 and Figure 8 were tabulated and statistically summarized.

To more clearly quantify the changes in bias, the average bias values in the horizontal and vertical directions before and after the improvements were statistically analyzed. For ease of comparison, Table 2 presents the absolute values of the average bias for the airborne data. From the table, it can be observed that the first dataset shows the most significant improvement in the horizontal direction, with a bias reduction of 16 cm. The fourth dataset shows a reduction of 7 cm, while the sixth dataset exhibits no change. The remaining datasets show improvements within 4 cm. In the vertical direction, the sixth dataset also shows no improvement, while the other datasets exhibit bias reductions ranging from 1 to 3 cm. Overall, the bias of the sixth dataset remains unchanged, while the other five datasets show varying degrees of reduction. These results indicate that the improved algorithm has a positive effect on enhancing accuracy.

Relying solely on bias statistics is insufficient to fully demonstrate the improvement in accuracy. To further validate whether the experimental platform proposed in this study enhances the positioning accuracy of UAVs, the RMSE in the horizontal and vertical directions before and after the improvements was tabulated and analyzed. Table 3 presents the statistical results of the RMSE for the airborne data. From Table 3, it can be observed that, except for the sixth dataset, all other datasets show varying degrees of reduction in RMSE in both the horizontal and vertical directions. The first dataset exhibits the most significant improvement in the horizontal direction, with an RMSE reduction of 58 cm. The third and fourth datasets also show notable improvements, with RMSE reductions of approximately 10 cm. The second dataset shows a minor improvement, with an RMSE reduction of only 2 cm. In the vertical direction, the improvement rates are relatively lower compared to the horizontal direction. Specifically, the second and sixth datasets show no significant improvement, while the first, third, fourth, and fifth datasets exhibit improvements, with the third dataset showing the best performance—an RMSE reduction of about 20 cm. 

**Figure 9:**
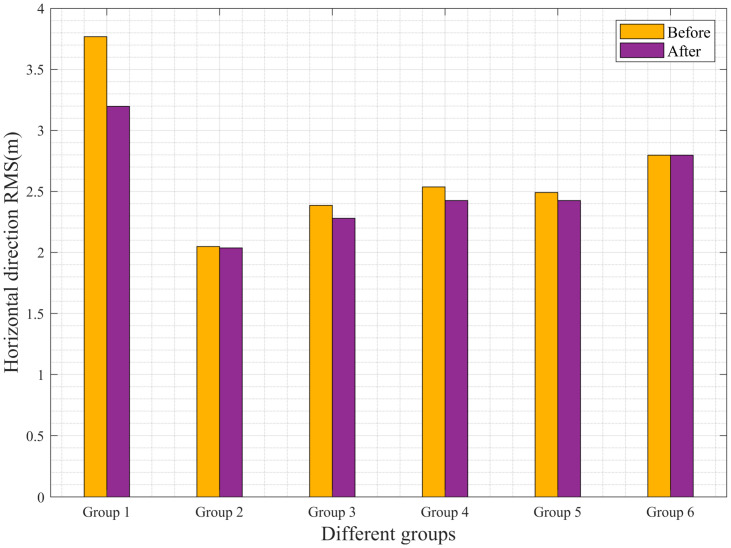
Histogram of RMSE in the horizontal direction of airborne data.

**Figure 10:**
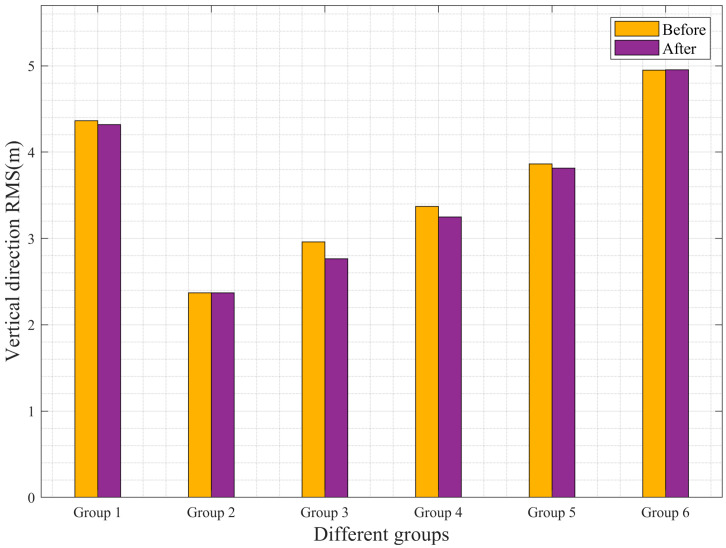
Histogram of RMSE in the elevation direction of airborne data.

To provide a more intuitive representation of the accuracy changes, histograms of the RMSE values before and after the improvements were generated. Figure 9 and Figure 10 show the RMSE histograms for the horizontal and vertical directions, respectively. From the figures, it is evident that, except for the sixth dataset, all other datasets show noticeable reductions in RMSE. The first dataset achieves a 15.38% improvement in the horizontal direction, while the third and fourth datasets show improvement rates of approximately 5%. In the vertical direction, the third dataset exhibits an improvement rate of about 6.76%. These results fully demonstrate that the improved loosely coupled integrated navigation platform achieves higher computational accuracy.

In summary, the real-time loosely coupled experimental platform used in this study is capable of normal data processing and computation. Compared to the original software, the proposed software platform demonstrates higher accuracy and stability in loosely coupled integration.

## 5. Conclusions

This paper presents a real-time GNSS/INS loosely coupled algorithm platform based on the open-source software KF_GINS. The algorithm platform supports real-time transmission and processing of raw GNSS and IMU data. Building upon the original software, we have integrated several enhanced features, including initial alignment based on GNSS solutions, anomaly detection for GNSS positioning results, and a Kalman filter model with position and velocity constraints. To validate the performance of the algorithm platform, multiple experiments were conducted using low-cost GNSS/INS devices for comprehensive verification and analysis. First, the positioning accuracy before and after the improvements was analyzed through vehicle-mounted experiments. The results demonstrate that the proposed platform can perform real-time loosely coupled solutions effectively. Subsequently, the improvement effects of the platform in UAV applications were verified through multiple experiments. It was found that the improved experimental software significantly outperforms the original software in terms of accuracy. The experimental results indicate the following:

(1) The proposed algorithm platform is capable of real-time wireless transmission and processing of raw GNSS and IMU data. By integrating GNSS velocity information, it effectively mitigates the drift caused by low-cost inertial sensors, thereby improving the positioning accuracy of the integrated navigation system.
(2) The improved software demonstrates enhanced positioning accuracy in both the horizontal and vertical directions. In the UAV experiments, the RMSE in the horizontal direction was reduced by up to 58 cm, representing an improvement rate of 15.38%. In the vertical direction, the RMSE was reduced by a maximum of 20 cm, with an improvement rate of 6.76%.

In subsequent experiments, we will focus on addressing challenges such as GNSS signal loss and initial attitude determination for UAVs. Additionally, other sensors, such as radar and cameras, can be integrated into the system to further enhance the accuracy of UAV positioning and attitude estimation. These multi-sensor fusion approaches are expected to improve the robustness and reliability of the navigation system, especially in complex environments where GNSS signals are weak or unavailable.

## Figures and Tables

**Table 1:** Parameters of the integrated navigation device.

Device		M22
gyroscope	Range (°/s)	±300 (XY)/±460 (Z)
	Bias stability (°/h)	5
accelerometer	Range (g)	±16
	Bias stability (µg)	50
GNSS receivers	System and frequency	BDS: B1I/B2I/B3I/B2a GPS: L1/L2/L5 GALILEO: E1/E5a/E5b GLONASS: L1/L2
Price (yuan)		¥8000

**Table 2:** Average bias statistics of airborne data.

Data	Bias (m)			
	Horizontal Direction		Vertical Direction	
	Before	After	Before	After
Group 1	2.98	2.82	2.38	2.37
Group 2	1.94	1.93	2.32	2.32
Group 3	2.27	2.23	2.74	2.71
Group 4	2.39	2.32	3.23	3.20
Group 5	2.23	2.19	3.61	3.60
Group 6	2.68	2.68	4.85	4.85

**Table 3:** Airborne data RMSE statistics.

Data	RMSE (m)			
	Horizontal Direction		Vertical Direction	
	Before	After	Before	After
Group 1	3.77	3.19	4.37	4.32
Group 2	2.05	2.03	2.37	2.36
Group 3	2.39	2.27	2.96	2.76
Group 4	2.54	2.43	3.37	3.24
Group 5	2.49	2.42	3.87	3.81
Group 6	2.80	2.79	4.95	4.95

## Data Availability

The data involved in this study can be obtained by contacting the corresponding author and will not be publicly disclosed at this time.
